# Supplementary material for: Branched actin networks are organized for asymmetric force production during clathrin-mediated endocytosis in mammalian cells
Source: Nat Commun. 2022 Jun 22;13:3578. doi: 10.1038/s41467-022-31207-5 (PMC9217951; doi:10.1038/s41467-022-31207-5)
Supplement: Supplementary file 1 — Supplementary Information [file 41467_2022_31207_MOESM1_ESM.pdf]

**Supplementary information**

# Branched actin networks are organized for asymmetric force production during clathrin-mediated endocytosis in mammalian cells

Meiyan Jin[1], Cyna Shirazinejad[1, 2], Bowen Wang[3], Amy Yan[1], Johannes Schöneberg[1, 4], Srigokul Upadhyayula[1], Ke Xu[3], David G. Drubin[1*]

[1] Department of Molecular and Cell Biology, University of California, Berkeley, CA 94720

[2] Biophysics Graduate Group, University of California Berkeley; Berkeley, CA, 94720

[3] Department of Chemistry, University of California, Berkeley, CA 94720

[4] Current address: Department of Pharmacology, and Department of Chemistry and Biochemistry, University of California, San Diego, CA 92093

These authors contributed equally: Meiyan Jin, Cyna Shirazinejad

Correspondence to DGD (drubin@berkeley.edu)

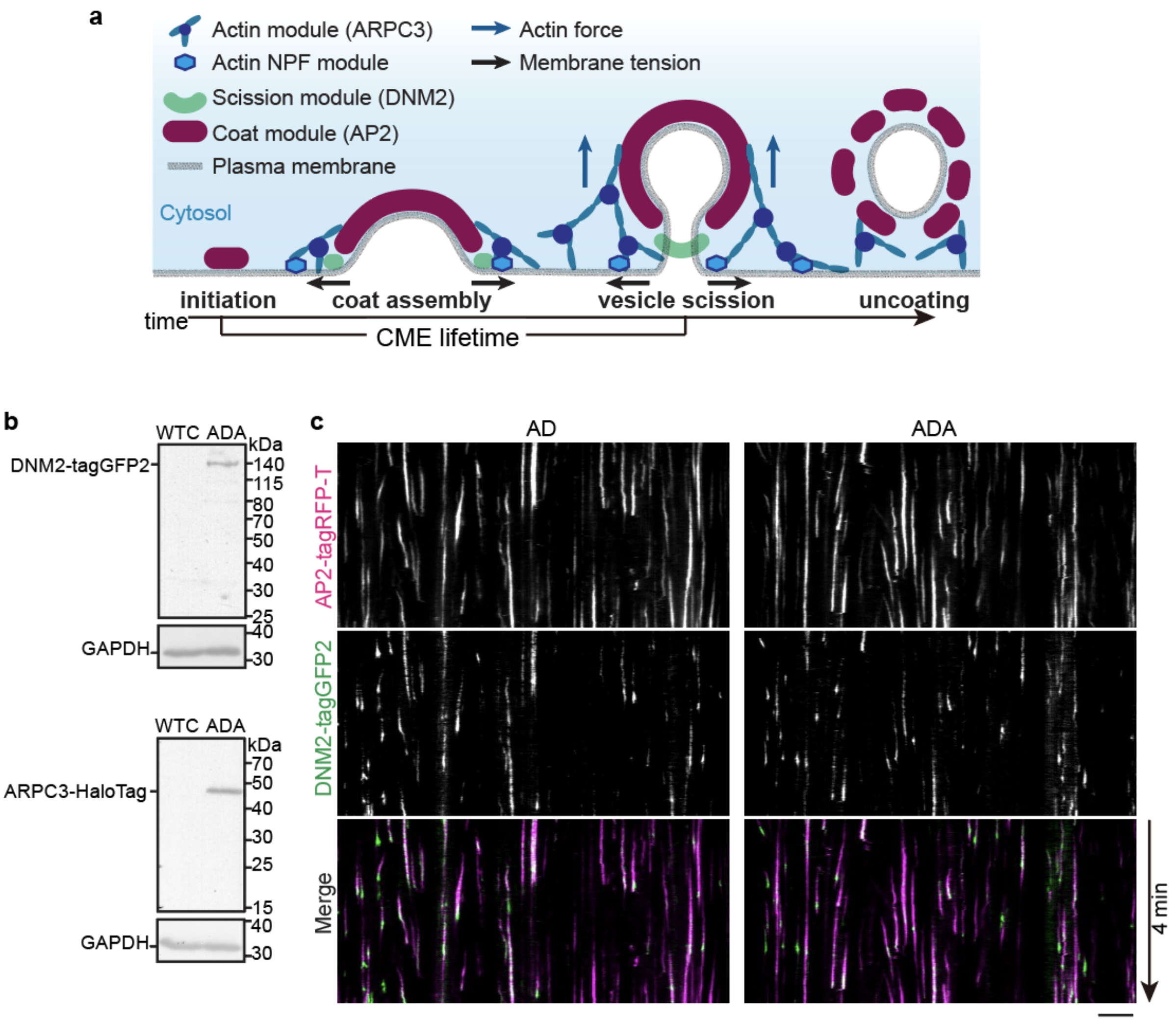


**Supplementary Fig. 1: Genome-edited iPSCs show dynamic CME sites.**

**a** Schematic model of CME. Mammalian CME proteins can be grouped into several modules, including the coat, WASP and Myosin / actin nucleation promoting factor (NPF), actin and scission modules. Actin networks provide pulling forces to invaginate the membrane against membrane tension. **b** Immunoblot analysis of cell extracts from the WT (WTC) and genome-edited (AP2M1-tagRFP-T/DNM2-tagGFP2/ARPC3-HaloTag; ADA) human iPSCs. The labeled proteins were detected with tag(CGY)FP, HaloTag, and GAPDH (loading control) antisera respectively. N=1. Uncropped and unprocessed scans of the blots are provided in the Source Data file. **c** Kymograph of representative CME sites of double-edited (AP2M1-tagRFP-

T/DNM2-tagGFP2; AD) and triple-edited (AP2M1-tagRFP-T/DNM2-tagGFP2/ARPC3-HaloTag; ADA) cells. Scale bar: 5µm.

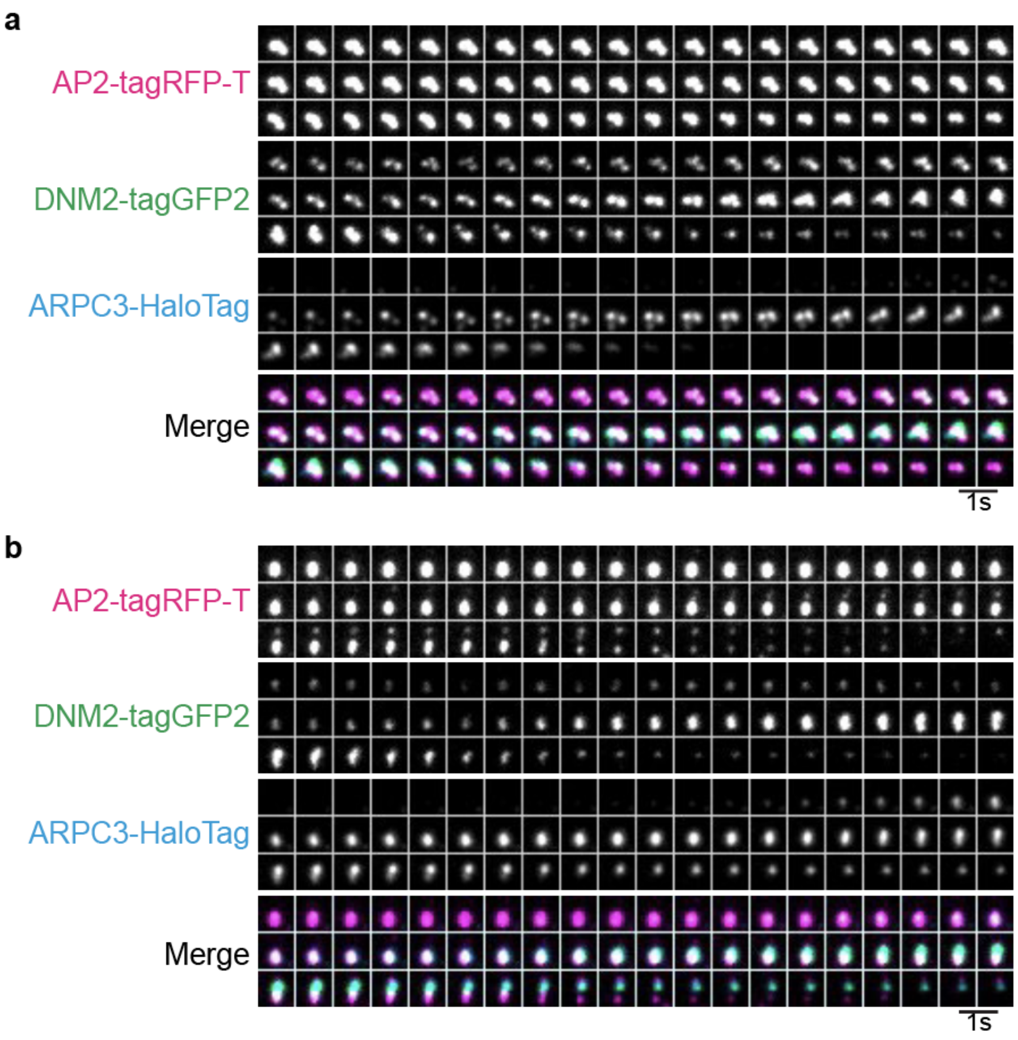


**Supplementary Fig. 2: Actin assembles at different types of CME sites.**

**a** Montage of a representative ARPC3 positive CME plaque from a TIRF movie of triple-edited (AP2M1-tagRFP-T/DNM2-tagGFP2/ARPC3-HaloTag; ADA) human iPSCs (Supplementary Movie 2). **b** Montage of a representative ARPC3 positive splitting CME site from a TIRF movie of triple-edited (AP2M1-tagRFP-T/DNM2-tagGFP2/ARPC3-HaloTag; ADA) human iPSCs (Supplementary Movie 2).

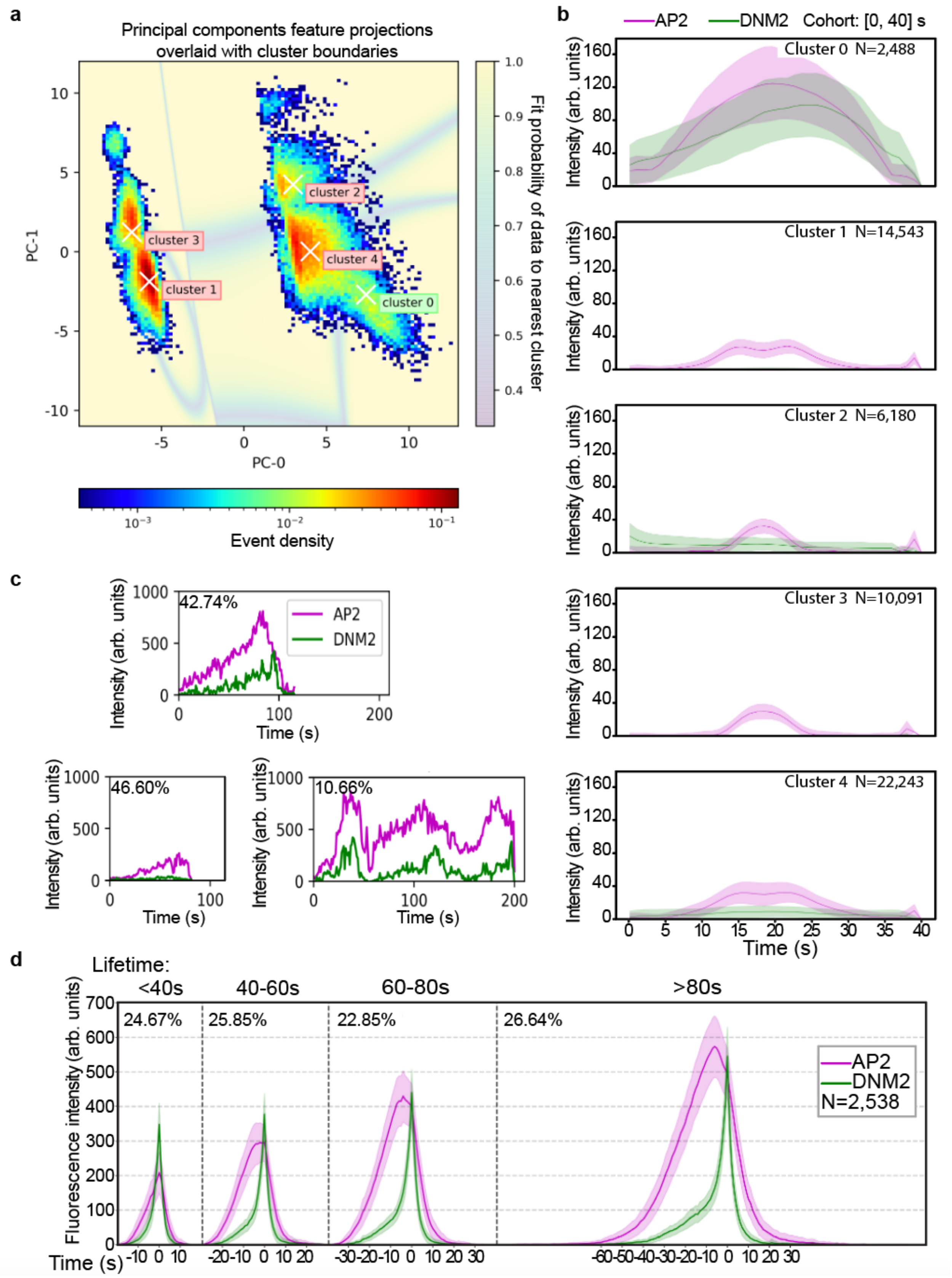
a
Principal components feature projections overlaid with cluster boundaries
cluster 0
cluster 1
cluster 2
cluster 3
cluster 4
PC-0
PC-1
Fit probability of data to nearest cluster
Event density
b
AP2
DNM2
Cohort: [0, 40] s
Cluster 0 N=2,488
Cluster 1 N=14,543
Cluster 2 N=6,180
Cluster 3 N=10,091
Cluster 4 N=22,243
Intensity (arb. units)
Time (s)
c
42.74%
46.60%
10.66%
Intensity (arb. units)
Time (s)
d
Lifetime:
<40s
40-60s
60-80s
>80s
24.67%
25.85%
22.85%
26.64%
N=2,538
Fluorescence intensity (arb. units)
Time (s)

**Supplementary Fig. 3: Filtering methods for selection of CME sites.**

**a** 2-D histogram of the first two principal components (PCs) of AP2 and DNM2 dynamic features. Of the 121,349 total tracks detected by CMEanalysis, ~49% were valids (as defined previously[30]), ~31% had detection gaps, ~15% were persistent events, and ~5% were split/merges. Only valid tracks (N=59,239), which appear and disappear during the course of movie acquisition, were used to generate filtering methods and for subsequent analysis. The shaded underlay represents simulated data points in principal component space and their individual probabilities of belonging to the nearest cluster center. Cluster 0 shows data points in the DNM2-positive cluster, which contains 10.02% of total valid tracks. Clusters 1-4 represented 24.60%, 10.43%, 17.03%, and 37.91% of valid tracks, respectively. **b** Cohort plots of the shortest AP2 events (<40 seconds) from each cluster. Cluster 0 represents DNM2-positive events where a strong DNM2 signal is detected. Data are presented as mean values +/- 1/4 standard deviation. **c** DNM2-positive events are sorted by the number of DNM2 peaks using a peak-detection scheme. Representative intensity vs time plots of a single-peaked event (top), non-peak event (bottom left) and a multi-peaked event (bottom right). Percentage of the number of the events in each class is shown next to the plot. **d** Single-peaked DNM2 events (N=2,538), hereon named CME sites, are grouped into lifetime cohorts and aligned to the peak of the DNM2 channel. Percentage of the number of the CME sites in each cohort is shown next to the plot. Data are presented as mean values +/- 1/4 standard deviation.

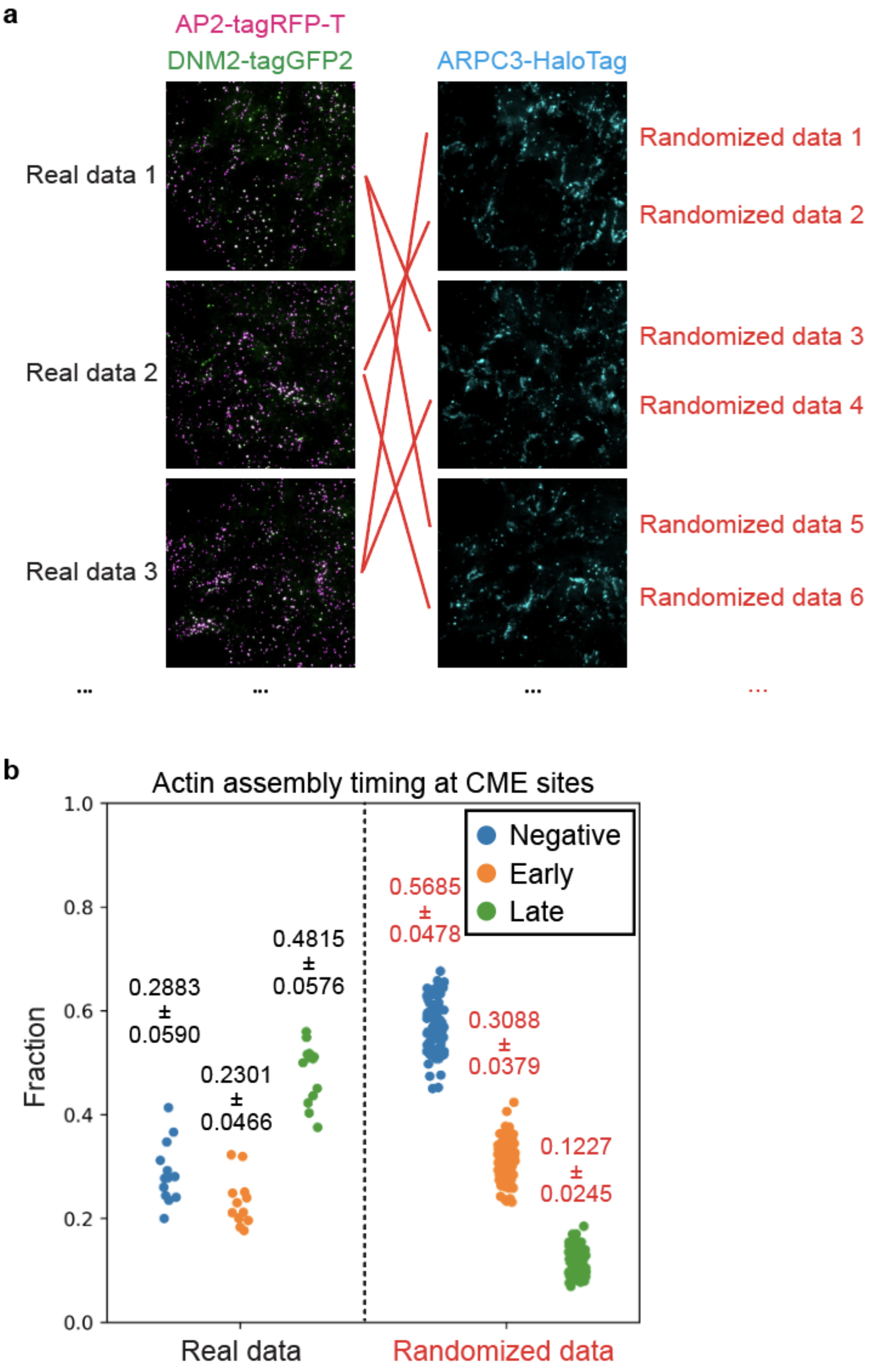
a
AP2-tagRFP-T
DNM2-tagGFP2
ARPC3-HaloTag
Real data 1
Real data 2
Real data 3
Randomized data 1
Randomized data 2
Randomized data 3
Randomized data 4
Randomized data 5
Randomized data 6
...
b
Actin assembly timing at CME sites
Negative
Early
Late
Fraction
1.0
0.8
0.6
0.4
0.2
0.0
0.2883 ± 0.0590
0.2301 ± 0.0466
0.4815 ± 0.0576
0.5685 ± 0.0478
0.3088 ± 0.0379
0.1227 ± 0.0245
Real data
Randomized data

**Supplementary Fig. 4: Computational analysis reveals actin network assembly at the late stage of CME.**

**a** We generated a randomized data set by pairing ARPC3 images with AP2 and DNM2 images from an unrelated movie. **b** The fraction of CME sites without actin assembly (Negative), with early actin assembly (ARPC3 signal disappears before DNM2 signal peak), and with late actin assembly (ARPC3 signal overlaps with DNM2 signal peak) were calculated for real data movies and randomized control movies (**a**). In the randomized dataset, we detected early "assembly" of actin in a similar fraction of CME events as in the real data set, indicating that presence of actin detected early in CME is very likely an artificial association caused when nearby actin structures overlap with CME sites by chance. Mean and standard deviation are shown on the graph. Source data are provided in the Source Data file.

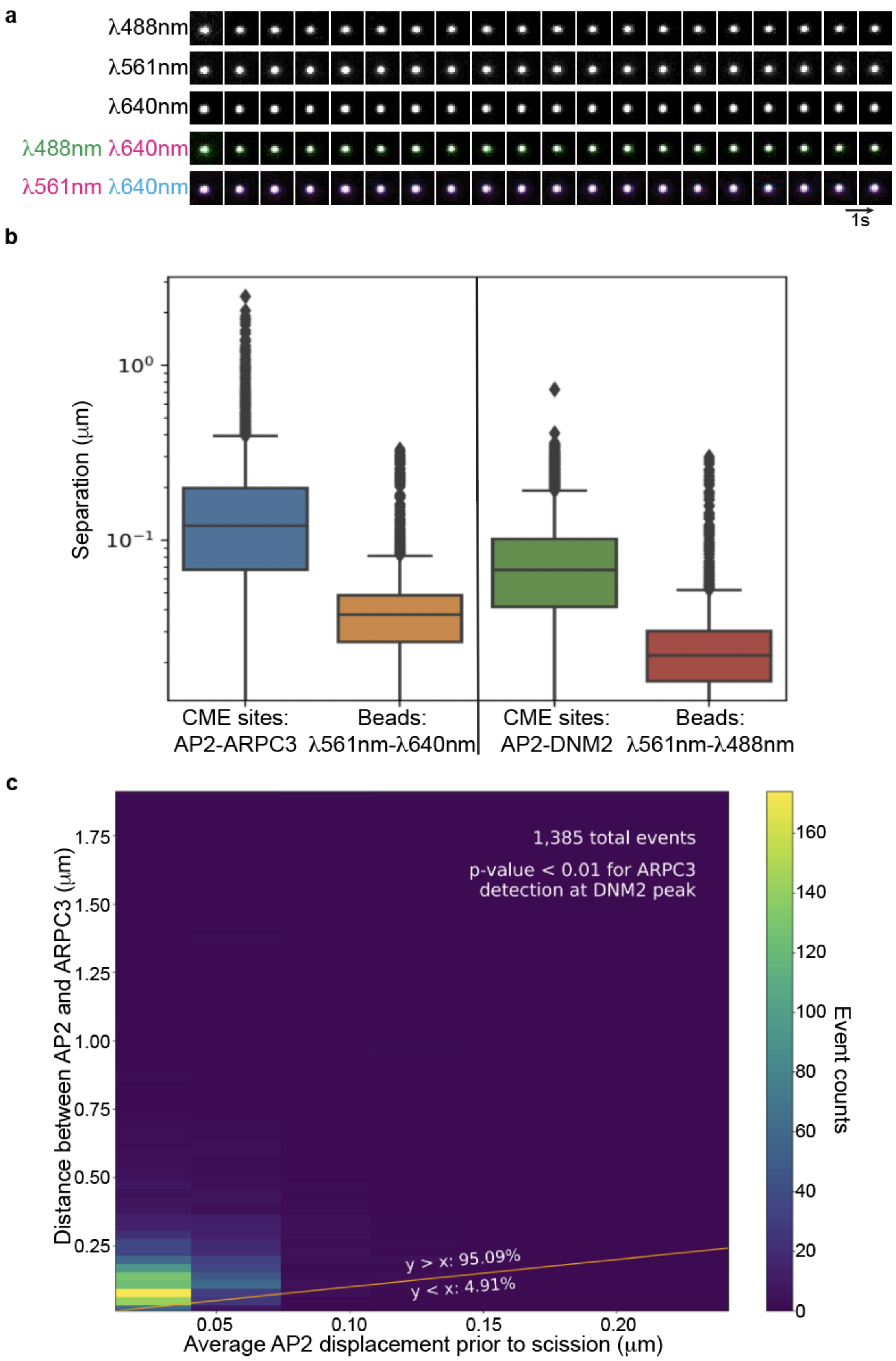
a
λ488nm
λ561nm
λ640nm
λ488nm λ640nm
λ561nm λ640nm
1s
b
Separation (μm)
$10^0$
$10^{-1}$
CME sites: AP2-ARPC3
Beads: λ561nm-λ640nm
CME sites: AP2-DNM2
Beads: λ561nm-λ488nm
c
1,385 total events
p-value < 0.01 for ARPC3 detection at DNM2 peak
y > x: 95.09%
y < x: 4.91%
Distance between AP2 and ARPC3 (μm)
Average AP2 displacement prior to scission (μm)
Event counts

**Supplementary Fig. 5: AP2-ARPC3 separation is not due to imaging artifacts.**

**a** Montage from a TIRF movie of a multi-fluorescence bead (Supplementary Movie 4). Size of field of view: 2µm x 2µm. Intervals: 1sec. **b** A boxplot of inter-channel distances (note: y-axis is base-10 log scale) between centroids of either CME sites or beads to highlight that the observed separation between marked proteins exceeds the measured chromatic aberration. Beads were compared between the same pairs of channels as used to image the tagged proteins. Box plot elements: center line, median; box limits, upper and lower quartiles; whiskers, 1.5x interquartile range; points, outliers. Source data are provided in the Source Data file. **c** A heat map graph of distance between AP2 and ARPC3 signals before scission, and average AP2 frame to frame displacement within 6 seconds before scission. Over 95% of the CME events present larger AP2-ARPC3 separation than AP2 displacement. N= 1,385.

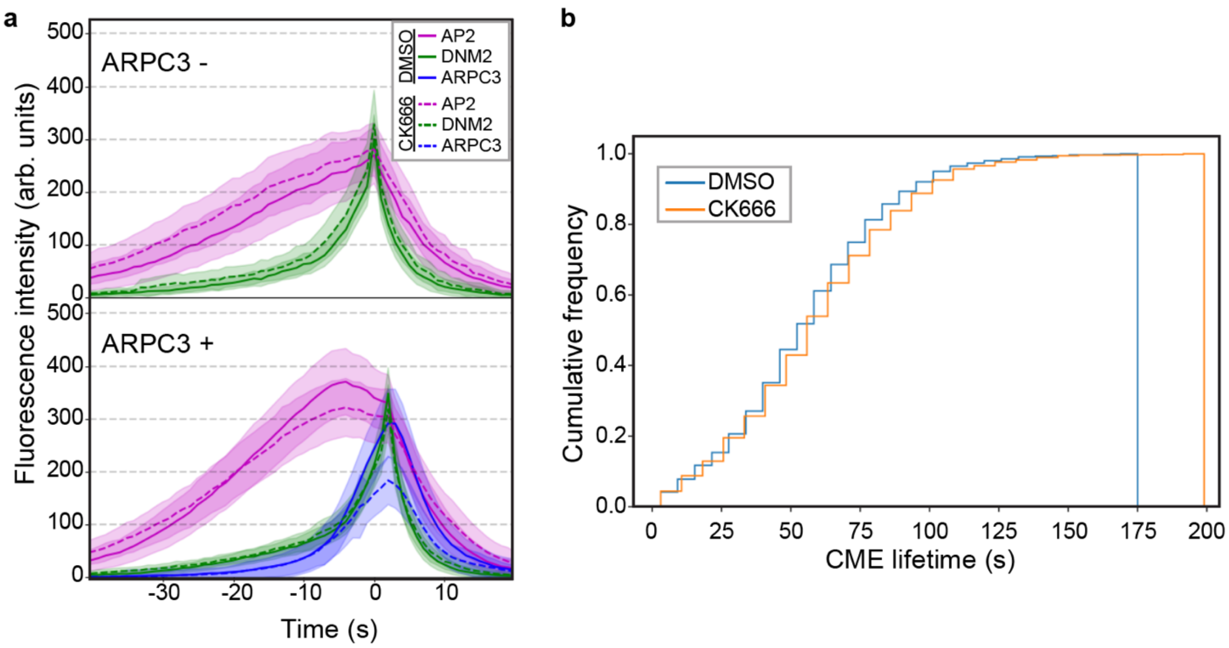


**Supplementary Fig. 6: Arp2/3-mediated actin assembly facilitates CME.**

**a** Intensity vs time plots of averaged ARPC3 negative (top) and positive (bottom) CME sites in ADA cells after 1-5min treatment with 2% DMSO (solid lines) or 100 µM CK666 (dashed lines). Events were aligned to the frames showing the maximum DNM2 intensity. Error bar: ¼ standard deviation. ARPC negative CME sites: DMSO: N=402, CK666: N=261, ARPC3 positive CME sites: DMSO: N=1,006, CK666: N=580. **b** CK666-treated cells have longer CME lifetimes than DMSO-treated control cells. p-value from two-sided Kolmogorov-Smirnov test: 1.83e-3. Source data are provided in the Source Data file.

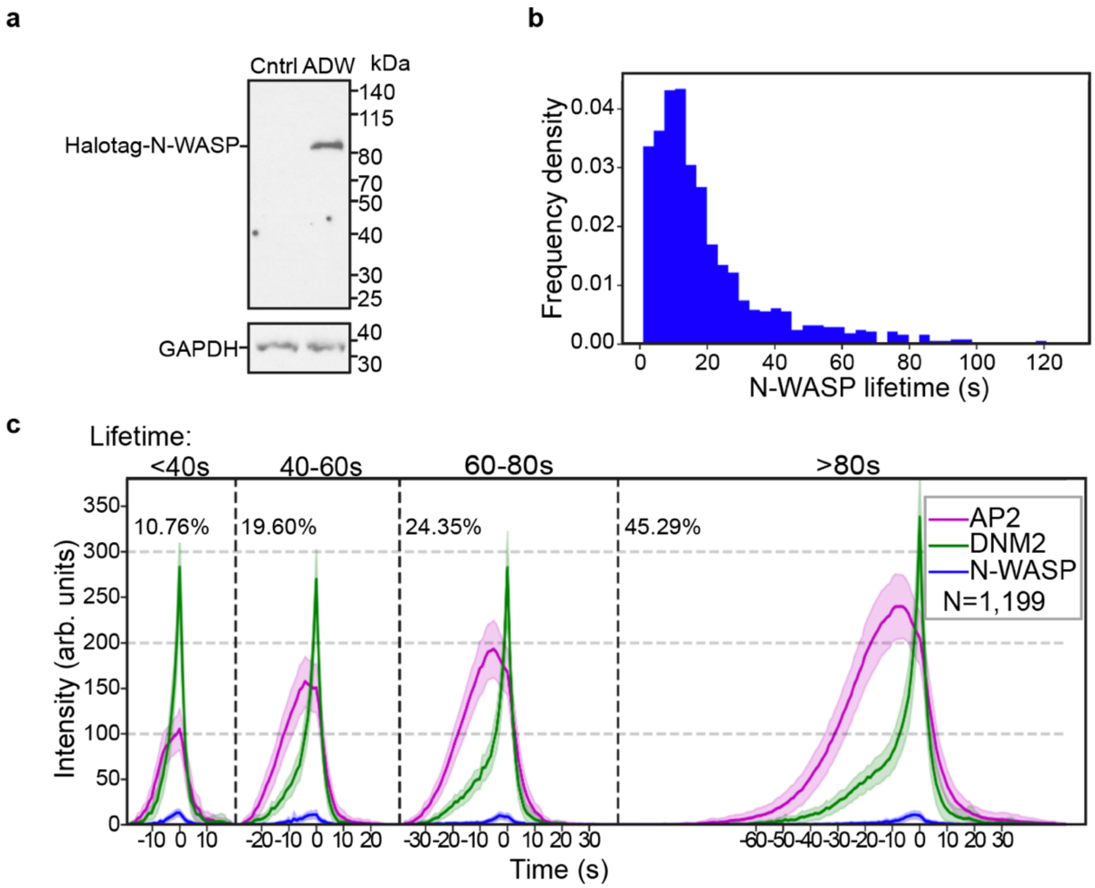


**Supplementary Fig. 7: Dynamics of N-WASP at CME sites.**

**a** Immunoblot analysis of cell extracts from the control and genome-edited (AP2M1-tagRFP-T/DNM2-tagGFP2/HaloTag-WASL; ADW) human iPSCs. The labeled proteins were detected with HaloTag and GAPDH (loading control) antisera respectively. N=1. Uncropped and unprocessed scans of the blots are provided in the Source Data file. **b** Histogram of N-WASP lifetime at CME sites. The lifetime is measured from the first frame of the N-WASP signal to the presumed scission time (the peak of DNM2 signal). **c** Intensity vs time plots of cohorts of N-WASP positive CME sites in ADW cells. Events are grouped into cohorts by the lifetimes of AP2 and aligned to the frames showing the maximum DNM2 intensity. Data are presented as mean values +/- 1/4 standard deviation. N=1,199. Source data are provided in the Source Data file.
