# Supplementary material for: Branched actin networks are organized for asymmetric force production during clathrin-mediated endocytosis in mammalian cells
Source: Nat Commun. 2022 Jun 22;13:3578. doi: 10.1038/s41467-022-31207-5 (PMC9217951; doi:10.1038/s41467-022-31207-5)
Supplement: Supplementary file 3 — Description of Additional Supplementary Files [file 41467_2022_31207_MOESM3_ESM.pdf]

Description of Additional Supplementary Files

File Name: Supplementary Movie 1
Description: A representative TIRF time-lapse movie (4min) of AP2M1-tagRFP-T (magenta) and DNM2-tagGFP2 (green) in AD cells. Scale bar: 5µm. Acquisition: 1 s/frame. Frame rate: 10 frames/s. Related to Supplementary Fig. 1.

File Name: Supplementary Movie 2
Description: A representative TIRF time-lapse movie (4min) of AP2M1-tagRFP-T (magenta), DNM2-tagGFP2 (green), and JF635 ligand-conjugated ARPC3- HaloTag (cyan) in ADA cells. Scale bar: 5µm. Acquisition: 1 s/frame. Frame rate: 10 frames/s. Related to Fig. 2 and Supplementary Fig. 1, 2.

File Name: Supplementary Movie 3
Description: A representative Airyscan time-lapse movie (3min) of DNM2-tagGFP2 (red) and JF635 ligand-conjugated ARPC3- HaloTag (cyan) on the ventral plasma membrane of ADA cells. The highlighted region is shown in Fig. 3a. Scale bar: 5µm. Acquisition: 0.2 s/frame. Frame rate: 50 frames/s. Related to Fig. 3.

File Name: Supplementary Movie 4
Description: A representative TIRF time-lapse movie (4min) of multi-fluorescence beads (488nm channel: green, 561nm channel: magenta, 640nm channel: cyan). Scale bar: 5µm. Acquisition: 1 s/frame. Frame rate: 10 frames/s. Related to Supplementary Fig. 5.

File Name: Supplementary Movie 5
Description: A representative TIRF time-lapse movie (4min) of AP2M1-tagRFP-T (magenta), DNM2-tagGFP2 (green) and JF635 ligand-conjugated HaloTagN-WASP (cyan) in ADW cells. Scale bar: 5µm. Acquisition: 1 s/frame. Frame rate: 10 frames/s. Related to Fig. 6.
