# Supplementary material for: Branched actin networks are organized for asymmetric force production during clathrin-mediated endocytosis in mammalian cells
Source: Nat Commun. 2022 Jun 22;13:3578. doi: 10.1038/s41467-022-31207-5 (PMC9217951; doi:10.1038/s41467-022-31207-5)
Supplement: Supplementary file 9 — Reporting Summary [file 41467_2022_31207_MOESM9_ESM.pdf]

Corresponding author(s): David G. Drubin

Last updated by author(s): May 23, 2022

Reporting Summary

Nature Portfolio wishes to improve the reproducibility of the work that we publish. This form provides structure for consistency and transparency in reporting. For further information on Nature Portfolio policies, see our Editorial Policies and the Editorial Policy Checklist.

Statistics

For all statistical analyses, confirm that the following items are present in the figure legend, table legend, main text, or Methods section.

n/a	Confirmed	
☐	☒	The exact sample size (*n*) for each experimental group/condition, given as a discrete number and unit of measurement
☒	☐	A statement on whether measurements were taken from distinct samples or whether the same sample was measured repeatedly
☐	☒	The statistical test(s) used AND whether they are one- or two-sided<br>*Only common tests should be described solely by name; describe more complex techniques in the Methods section.*
☒	☐	A description of all covariates tested
☒	☐	A description of any assumptions or corrections, such as tests of normality and adjustment for multiple comparisons
☒	☐	A full description of the statistical parameters including central tendency (e.g. means) or other basic estimates (e.g. regression coefficient) AND variation (e.g. standard deviation) or associated estimates of uncertainty (e.g. confidence intervals)
☐	☒	For null hypothesis testing, the test statistic (e.g. *F*, *t*, *r*) with confidence intervals, effect sizes, degrees of freedom and *P* value noted<br>*Give P values as exact values whenever suitable.*
☒	☐	For Bayesian analysis, information on the choice of priors and Markov chain Monte Carlo settings
☒	☐	For hierarchical and complex designs, identification of the appropriate level for tests and full reporting of outcomes
☒	☐	Estimates of effect sizes (e.g. Cohen's *d*, Pearson's *r*), indicating how they were calculated

Our web collection on statistics for biologists contains articles on many of the points above.

Software and code

Policy information about availability of computer code

Data collection	Nikon NIS-Elements was used to collect TIRF live-cell imaging data. Zen 3.1 was used to collect Airyscan live-cell imaging data.
Data analysis	ImageJ, Prism 9.3.1, Insight3 V4.28.10, MATLAB_2018b and Python 3.6 were used for data analysis.

For manuscripts utilizing custom algorithms or software that are central to the research but not yet described in published literature, software must be made available to editors and reviewers. We strongly encourage code deposition in a community repository (e.g. GitHub). See the Nature Portfolio guidelines for submitting code & software for further information.

Data

Policy information about availability of data

All manuscripts must include a data availability statement. This statement should provide the following information, where applicable:

- Accession codes, unique identifiers, or web links for publicly available datasets
- A description of any restrictions on data availability
- For clinical datasets or third party data, please ensure that the statement adheres to our policy

All the raw data used for analysis can be found at https://drive.google.com/drive/folders/19t4J4nRg9MEVq0BjL1_YjbblBObO5EcB?usp=sharing .

Field-specific reporting

Life sciences study design

All studies must disclose on these points even when the disclosure is negative.

Sample size	For the quantitative analysis of TIRF live-cell imaging, each cell lines were imaged from at least two sets of independently prepared cell samples. For each sample, at least three non-overlapping regions which contain at least three cells per imaging region were imaged. Over a thousand of CME events were analyzed for each cell line.<br>For the analysis of Airyscan live-cell imaging, cells were imaged from three sets of independently prepared cell samples. For each sample, at least three non-overlapping regions were imaged.<br>For STORM fixed cell imaging, at least three different cells were imaged for each conditions.
Data exclusions	The particle tracking software filters events based on criteria described in detail in the legend to Supplemental Figure 3.
Replication	At least three biological replicates were performed for all imaging experiments. PCR, sequencing and Western blotting experiments for cell line verifications were performed once.
Randomization	The regions of imaging were randomly selected.
Blinding	We applied automatic computational analysis without any human selection of samples, therefore, blinding was not necessary.

Reporting for specific materials, systems and methods

We require information from authors about some types of materials, experimental systems and methods used in many studies. Here, indicate whether each material, system or method listed is relevant to your study. If you are not sure if a list item applies to your research, read the appropriate section before selecting a response.

Materials & experimental systems

n/a	Involved in the study	
☐	☒	Antibodies
☐	☒	Eukaryotic cell lines
☒	☐	Palaeontology and archaeology
☒	☐	Animals and other organisms
☒	☐	Human research participants
☒	☐	Clinical data
☒	☐	Dual use research of concern

Methods

n/a	Involved in the study	
☒	☐	ChIP-seq
☒	☐	Flow cytometry
☒	☐	MRI-based neuroimaging

Antibodies

Antibodies used	Tag(CGY)FP: Evrogen, Cat#: AB121; HaloTag: Promega, Cat#: G9211; GAPDH: Proteintech, Cat#: 10494-1-AP; Clathrin light chain: Invitrogen, Cat#: MA5-11860.<br>Secondary antibodies: Donkey anti-mouse secondary-AF647: Thermo Fisher, Cat#: A32787; Donkey anti-mouse antibody: Jackson ImmunoResearch, Code#: 715-005-151; Donkey anti-rabbit antibody: Jackson ImmunoResearch, Code#: 711-005-152
Validation	Tag(CGY)FP: Statement on the manufacturer's website: The antibody was selected to recognize both denatured and native TagCFP, TagGFP, TagGFP2, TagYFP, PS-CFP2, AceGFP1, Case12, and HyPer. The antibody also recognizes EGFP.<br>HaloTag: Statements on the manufacturer's website: Can detect as low as 0.5–1ng of HaloTag fusion protein by Western blot.<br>GAPDH: Statements on the manufacturer's website: Antibody targets GAPDH in WB, RIP, IP, IHC, IF, FC, CoIP,ELISA applications and shows reactivity with human, mouse, rat, pig, arabidopsis , corn , cabbage, rice samples.<br>Clathrin light chain: Statements on the manufacturer's websites: Antibody targets Clathrin Light Chain in IF, IHC (P), IP, and WB applications and shows reactivity with Bovine and Human samples. Antibody specificity was demonstrated by siRNA mediated knockdown of target protein.<br>Donkey anti-mouse secondary-AF647: Statements on the manufacturer's websites: This antibody binds to heavy chains on mouse IgG and light chains on all mouse immunoglobulins. This antibody does not bind non-immunoglobulin mouse serum proteins or IgG from bovine, chicken, goat, guinea pig, hamster, horse, human, rabbit, rat or sheep.<br>Donkey anti-mouse antibody: Statements on the manufacturer's websites: Based on immunoelectrophoresis and/or ELISA, the antibody reacts with whole molecule mouse IgG. It also reacts with the light chains of other mouse immunoglobulins. No antibody was detected against non-immunoglobulin mouse serum proteins.<br>Donkey anti-rabbit antibody: Statements on the manufacturer's websites: Based on immunoelectrophoresis and/or ELISA, the antibody reacts with whole molecule rabbit IgG. It also reacts with the light chains of other rabbit immunoglobulins. No antibody was detected against non-immunoglobulin serum proteins.

Eukaryotic cell lines

Policy information about cell lines

Cell line source(s)	The WTC10 hiPSC parental line was obtained from the Bruce Conklin Lab at the University of California, San Francisco. All the cell lines used in the study, AD, ADA and ADW cells, were generated by genome-editing of WTC10 hiPSC line by the authors.
Authentication	All the cell lines used by this study were verified by genomic DNA purification, western blotting and Sanger sequencing and were authenticated by University of California, Berkeley Cell Culture Facility using short tandem repeat (STR) analysis.
Mycoplasma contamination	All the cell lines used in the study were tested negative for mycoplasma contamination.
Commonly misidentified lines (See ICLAC register)	No commonly misidentified lines were used in this study.
